# Hyaluronan Production by Renomedullary Interstitial Cells: Influence of Endothelin, Angiotensin II and Vasopressin

**DOI:** 10.3390/ijms18122701

**Published:** 2017-12-13

**Authors:** Sara Stridh, Fredrik Palm, Tomoko Takahashi, Mayumi Ikegami-Kawai, Malou Friederich-Persson, Peter Hansell

**Affiliations:** 1 Department of Medical Cell Biology, Uppsala University, Biomedical Center, SE-75123 Uppsala, Sweden; strs@rkh.se (S.S.); fredrik.palm@mcb.uu.se (F.P.); malou.friederich@mcb.uu.se (M.F.-P.); 2 Department of Health Sciences, Red Cross University College, SE-14152 Stockholm, Sweden; 3 Faculty of Pharmaceutical Sciences, Hoshi University, Tokyo 142-8501, Japan; tomoko-takahashi@mue.biglobe.ne.jp (T.T.); m-kawai@hoshi.ac.jp (M.I.-K.)

**Keywords:** hyaluronan, kidney, interstitium, medulla, endothelin, vasopressin, angiotensin II

## Abstract

The content of hyaluronan (HA) in the interstitium of the renal medulla changes in relation to body hydration status. We investigated if hormones of central importance for body fluid homeostasis affect HA production by renomedullary interstitial cells in culture (RMICs). Simultaneous treatment with vasopressin and angiotensin II (Ang II) reduced HA by 69%. No change occurred in the mRNA expressions of hyaluronan synthase 2 (HAS2) or hyaluronidases (Hyals), while Hyal activity in the supernatant increased by 67% and CD44 expression reduced by 42%. The autocoid endothelin (ET-1) at low concentrations (10^−10^ and 10^−8^ M) increased HA 3-fold. On the contrary, at a high concentration (10^−6^ M) ET-1 reduced HA by 47%. The ET-A receptor antagonist BQ123 not only reversed the reducing effect of high ET-1 on HA, but elevated it to the same level as low concentration ET-1, suggesting separate regulating roles for ET-A and ET-B receptors. This was corroborated by the addition of ET-B receptor antagonist BQ788 to low concentration ET-1, which abolished the HA increase. HAS2 and Hyal2 mRNA did not alter, while Hyal1 mRNA was increased at all ET-1 concentrations tested. Hyal activity was elevated the most by high ET-1 concentration, and blockade of ET-A receptors by BQ123 prevented about 30% of this response. The present study demonstrates an important regulatory influence of hormones involved in body fluid balance on HA handling by RMICs, thereby supporting the concept of a dynamic involvement of interstitial HA in renal fluid handling.

## 1. Introduction

Hyaluronan (HA) is a negatively charged interstitial glycosaminoglycan with large water attracting ability [1]. In the kidney, HA is predominantly found in the inner medulla during normal physiological conditions [2,3,4,5]. This site is responsible for the fine tuning of fluid electrolyte balance primarily under the influence of hormones such as angiotensin II (Ang II), aldosterone and vasopressin. The differential intrarenal distribution of HA is important for urine concentration and dilution [6]. We have previously demonstrated that during acute hydration medullary HA increases and, in contrast, HA content decreases during water deprivation [2,3]. It has been suggested that apart from the changes occurring in vasopressin-regulated aquaporins, the physicochemical properties of the interstitial matrix changes in relation to hydration status, which influences primarily water permeability [6]. Ginetzinsky [7] demonstrated that the HA-degrading enzyme hyaluronidase (Hyal) is excreted in the urine in larger amounts during dehydration and drops to virtually zero during hydration. It was suggested that the interstitial matrix was altered by Hyal activity, thereby changing the level of diuresis. Under pathological conditions of altered kidney function, HA is inappropriately regulated [6]. During renal ischemia-reperfusion injury or tubulointerstitial inflammation, renal cortical levels of HA increase, while medullary levels are largely unaltered, which may contribute to the pathophysiology of the disease process [4,8,9]. Indeed, suppression of HA accumulation during renal ischemia-reperfusion improves renal function, suggesting a protecting effect against ischemic insults [10]. During diabetes, both cortical and medullary levels are elevated [6], which may contribute to the phenotype due to the pro-inflammatory and water-attracting properties of HA.

We have previously demonstrated that a major contributor of interstitial HA in renal medulla is the renomedullary interstitial cell (RMIC) [11,12]. These cells express receptors for hormones and autocoids known to be involved in the regulation of fluid and electrolyte balance [13]. These receptors include Ang II AT1, vasopressin V1a, endothelin (ET)-A and -B, and bradykinin B2. RMICs in culture produce less HA during hyperosmotic conditions than during iso- and hypo-osmotic conditions [11,12], supporting the in vivo observations that HA content decreases during dehydration, while it increases during hydration [2,3].

Despite the observations from our and other laboratories that renal HA content changes during physiological and pathophysiological conditions, the mechanisms regulating HA content under these conditions are unclear. Thus, the aim of the present study was to determine the effects of hormones and an autocoid involved in normal regulation of body fluid balance on HA turnover by RMICs in culture. Furthermore, the importance of Hyal activity and of the HA scavenging receptor CD44 expression [14,15] for HA turnover were also investigated.

## 2. Results

### 2.1. Hyaluronan (HA) in Supernatant

**Figure 1:**
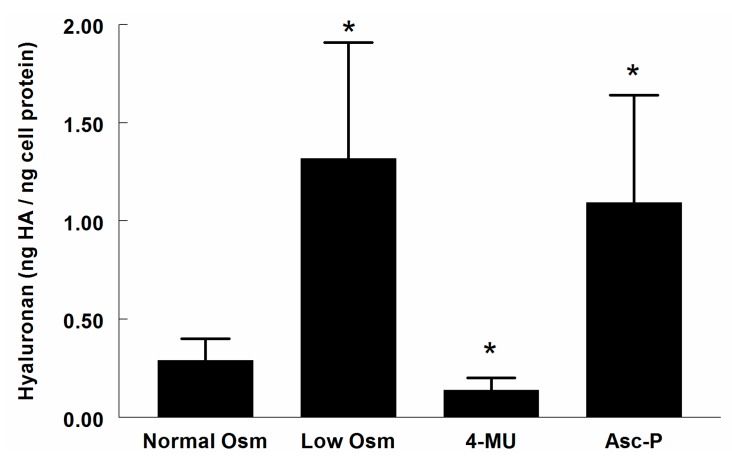
Hyaluronan (HA) content in supernatants of cultured renomedullary interstitial cells (RMICs) during control conditions (normal osmolality) and after 24 h exposure to hypo-osmotic media conditions (200 mOsm/kg H_2_O), a hyaluronidase inhibitor (Asc-P) or the HA synthesis inhibitor 4-MU.* *p* < 0.05 vs. corresponding value of control cells (normal osmolality).

The HA content in the supernatant of RMICs grown under iso-osmotic conditions was 0.29 ± 0.11 ng HA/ng cell protein (Figure 1). Changing growth media to hypo-osmotic conditions resulted in a more than 4-fold elevation of the HA content (*p* < 0.05). A similar increase occurred after treatment with the Hyal inhibitor l-ascorbic acid 6-hexadecanoate (Asc-P) (more than 3-fold elevation, *p* < 0.05), while the HA synthesis inhibitor 4-methylumbelliferone (4-MU) reduced the HA content by 52% (*p* < 0.05).

**Figure 2:**
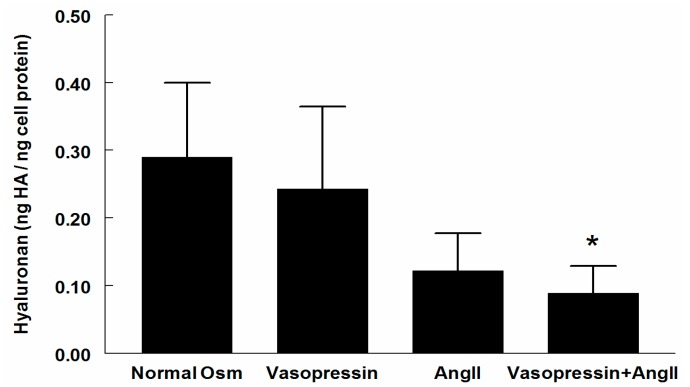
Hyaluronan (HA) content in the supernatant of cultured RMICs during control conditions (normal osmolality) and after 24 h exposure to angiotensin II (Ang II, 10^−6^ M), vasopressin (10^−6^ M), and a combination of Ang II and vasopressin. * *p* < 0.05 vs. corresponding value of control cells (normal osmolality).

Neither Ang II nor vasopressin alone reduced HA in the supernatant significantly (−16% and −58%, respectively, ns). However, when the combination of Ang II and vasopressin was used, HA was reduced by 69% (*p* < 0.05) (Figure 2).

**Figure 3:**
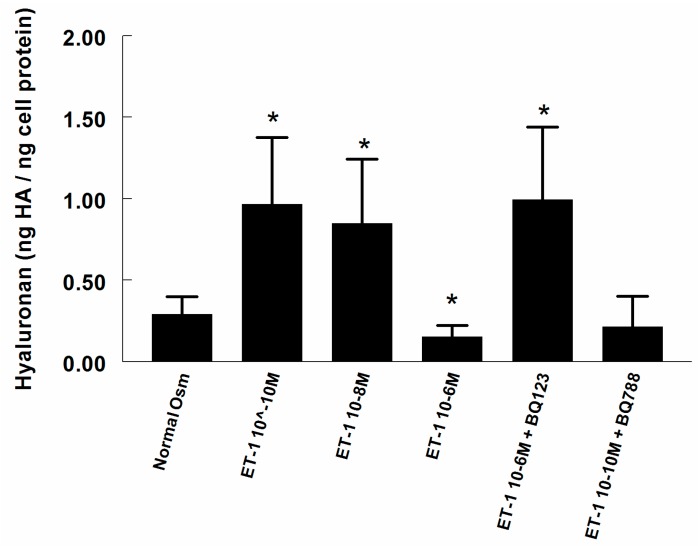
Hyaluronan (HA) content in the supernatant of cultured RMICs during control conditions and after 24 h exposure to endothelin (ET-1), with or without the ET-A receptor antagonist BQ123 or the ET-B receptor antagonist BQ788. * *p* < 0.05 vs. corresponding value of control cells (normal osmolality).

Endothelin-1 (ET-1) at 10^−10^ M and 10^−8^ M increased supernatant HA more than 3-fold (*p* < 0.05), while, on the contrary, a high concentration of ET-1 (10^−6^ M) reduced HA by 47% (*p* < 0.05, Figure 3) as compared with untreated control cells. The ET-A receptor antagonist BQ123 not only reversed the reducing effect of the high concentration of ET-1 on HA, but elevated it to the same level as low concentration ET-1 (10^−10^ M), suggesting an important mechanism involving the still active ET-B receptor. This was corroborated by the addition of ET-B receptor antagonist BQ788 to low concentration ET-1, which abolished the increase in HA.

### 2.2. Hyaluronidase (Hyal) Activity in Supernatant

**Figure 4:**
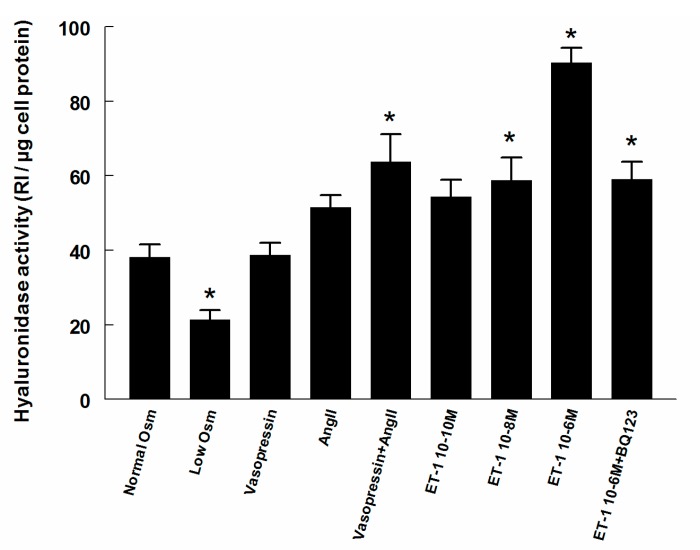
Hyaluronidase (Hyal) activity in supernatants of cultured RMICs during different treatments. Values are related to the amount of total cell protein in each culture dish. RI, relative intensity. * *p* < 0.05 vs. control cells (normal osmolality).

The Hyal activity in the supernatant of cells grown in low-osmolality media was 44% lower (*p* < 0.05) compared to during iso-osmotic conditions (Figure 4). Neither Ang II nor vasopressin alone altered the activity. However, the combination of Ang II and vasopressin increased the Hyal activity by 67% (*p* < 0.05). ET-1 increased the supernatant activity at 10^−8^ M and 10^−6^ M. At 10^−8^ M the activity increased by 54%, while the high concentration (10^−6^ M) elevated the Hyal activity by 137%. When the ET-A receptor antagonist BQ123 was added, the elevation in activity after the high concentration of ET-1 (10^−6^ M) was reduced and thus similar to that of the lower concentrations used (55%).

### 2.3. Hyaluronan Synthase (HAS) and Hyaluronidase mRNA in Renomedullary Interstitial Cells in Culture (RMICS)

**Figure 5:**
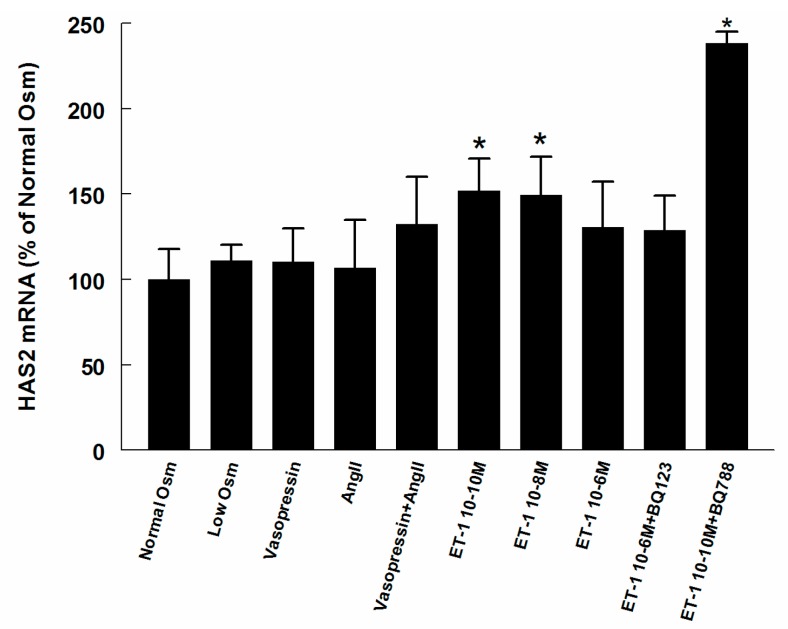
mRNA expressions of hyaluronan synthase 2 (HAS2) in RMICs during different treatments. All values are in relation to cells grown at normal osmolality = 100%. * *p* < 0.05 vs. control cells (normal osmolality).

**Figure 6:**
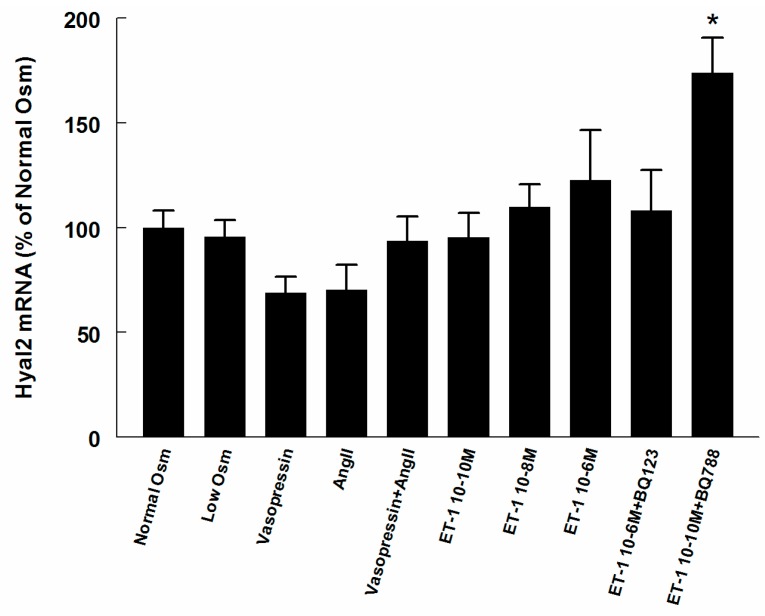
mRNA expressions of hyaluronidase 2 (Hyal 2) in RMICs during different treatments. All values are in relation to cells grown at normal osmolality = 100%. * *p* < 0.05 vs. control cells (normal osmolality).

HAS2 and Hyal2 mRNA expression did not change in most of the treatment groups (Figure 5 and Figure 6), however, HAS2 was increased in ET-1 groups of low and medium concentration, and BQ788 together with low concentration ET-1 increased expression of both HAS2 and Hyal2. 

**Figure 7:**
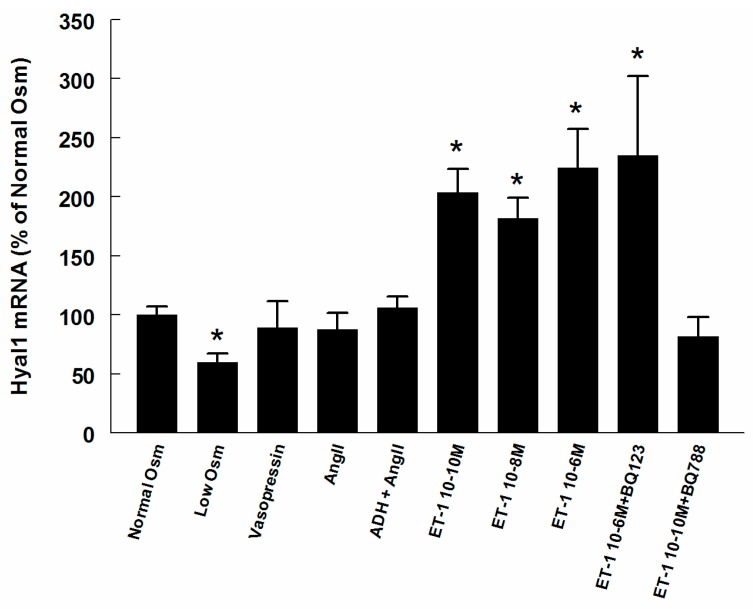
mRNA expressions of hyaluronidase 1 (Hyal 1) in RMICs during different treatments. All values are in relation to cells grown at normal osmolality = 100%. * *p* < 0.05 vs. control cells (normal osmolality).

In RMICs grown in hypo-osmolar conditions the Hyal1 mRNA was reduced by 35% as compared to iso-osmolar conditions (*p* < 0.05, Figure 7), while Ang II and vasopressin, alone or in combination, did not change the expression. All concentrations of ET-1 elevated the Hyal1 mRNA expression similarly. This elevation was not affected by the ET-A receptor blocker BQ123, but was abolished by the ET-B receptor blocker BQ788.

### 2.4. CD44 on RMIC Surface

**Figure 8:**
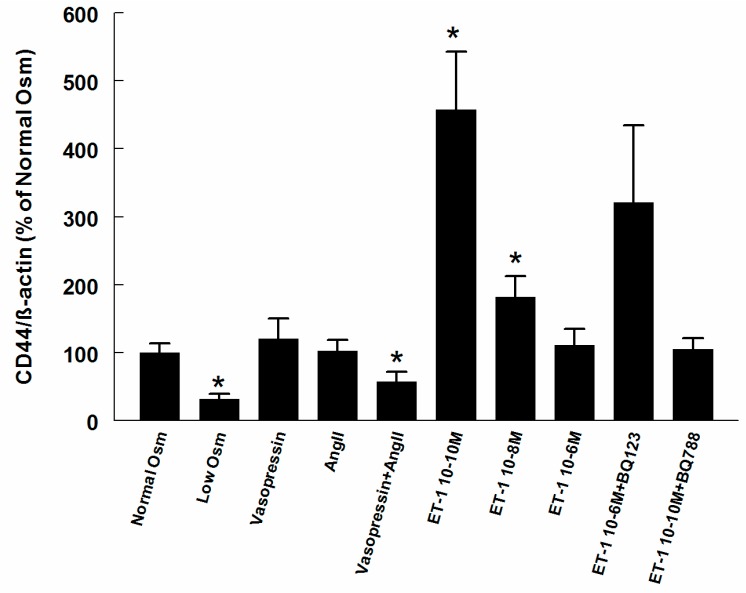
Expression of the scavenging receptor CD44 on the cell surface of RMICs during different treatments. All values are in relation to cells grown at normal osmolality = 100%. * *p* < 0.05 vs. control cells (normal osmolality).

The CD44 surface expression on RMICs grown in hypo-osmolar conditions was 68% lower than that on cells grown at iso-osmolar conditions (*p* < 0.05, Figure 8). No change in expression occurred by Ang II or vasopressin separately, but when combined the CD44 expression was reduced by 42% (*p* < 0.05). The lowest concentration of ET-1 (10^−10^ M) increased CD44 expression more than 4-fold (*p* < 0.05). The intermediate concentration of ET-1 (10^−8^ M) also increased the CD44 expression, but to a lesser extent. Low concentration of ET-1 in combination with ET-B receptor blocker BQ788 normalized CD44 expression. The higher concentration of ET-1 together with the ET-A receptor blocker BQ123 tended to increase CD44 expression, suggesting an effect over ET-B receptors.

## 3. Discussion

The present study demonstrates an important regulatory role of hormones involved in body fluid balance on HA turnover by cultured RMICs. Furthermore, the results demonstrate an important mechanism over altered Hyal activity to regulate HA turnover, which provides a rapid way to change HA as opposed to primarily regulating synthesis. Our previous studies have suggested an important role for HA in renomedullary water handling. During acute hydration the rat medullary interstitial HA content increases, while the opposite occurs during water deprivation [2,3]. The elevation in medullary interstitial HA content during excess water intake will antagonize medullary water reabsorption by changing the interstitial matrix properties, thereby resulting in reduced fluid conductance. The opposite occurs during water deprivation in conjunction with increased vasopressin-regulated aquaporins. Our present findings widen our understanding on how the composition of the renomedullary interstitial matrix changes in response to hydration status and also sets focus on hyaluronidases in order to achieve rapid responses.

Changes in overall HA content are due to changes in HA synthesis and/or HA degradation. The present study suggests that regulation of the degradation pathway is of major importance for the changes seen in supernatant HA both after a hypo-osmolar challenge as well as after hormonal action. The mRNA expression of HAS2 and Hyal2 in RMICs did not change after hypo-osmotic challenge, Ang II, or vasopressin, suggesting a change in Hyal activity. Our previous in vivo data in rats show no changes in the mRNA levels of HAS or Hyals after 2 h hydration when medullary HA is elevated [16], again suggesting a change in the activity. In the case of ET-1, mRNA levels of Hyal1 were elevated, while hypo-osmolality reduced the Hyal1 expression, which strengthens the proposition that the degradation pathway is an important way to change HA. It is of interest in this context that lack of hyaluronidases exacerbates renal post-ischemic injury, inflammation, and fibrosis, pointing to an ongoing defense mechanism [17].

The mechanism(s) underlying Hyal activation in the present study are not known but it is generally accepted that mRNA levels may not reflect activity or protein levels. Changing activity would be a more rapid way to increase catabolic function as opposed to primarily producing more of the enzyme *per se*. In a study by Albeiroti and colleagues [18] on platelets, it was demonstrated that Hyal2 is activated by being transported from intracellular sites to the surface of the cell, thereby achieving catabolic activity. This activation in an embryonic kidney cell line (as opposed to that in platelets) requires coexistence with CD44 expression on the plasma membrane [19]. In the present study on RMICs, we demonstrate that CD44 surface expression decreases during hypo-osmolar conditions of the media and lowers Hyal activity, which results in elevated amounts of HA in the media, thus suggesting reduced catabolic and internalization processes. The same line of reasoning regarding effects of Hyal activity and CD44 expression cannot be performed after treatment with Ang II, vasopressin, or ET-1, thus suggesting another pathway of Hyal activation which may include intracellular calcium and IP3 signaling [13].

In a previous in vitro study [11] in cultured rat RMICs, we found that the HA binding receptor CD44 is downregulated under hypo-osmotic conditions (mimicking in vivo hydration), while it is upregulated under hyperosmotic conditions (mimicking water deprivation). This was corroborated by the present study, which showed reduced CD44 expression when reducing growth media osmolality. Once HA binds to CD44 it can be internalized and degraded [14,20,21]. Furthermore, in an in vitro study [22] it was demonstrated that low ionic strength inhibits HA hydrolysis catalyzed by Hyal. This would imply that during hypo-osmotic conditions when low ionic strength applies both the cellular uptake of HA over CD44 and the breakdown of HA by Hyal, is reduced. The acute hydration-induced elevation in HA in vivo (within 2 h) [3] suggests an important role over inhibition of Hyals and not primarily increased HAS expression or activity.

ET-1 resulted in a biphasic response on supernatant HA levels which was related to agonist concentration. Such a concentration dependent biphasic response has previously been described for ET-1 on the effect on vascular smooth muscle (i.e., low concentration results in dilation and high concentration results in contraction) [23,24]. In the present study, low concentration ET-1 increased HA in the supernatant, while the high concentration reduced HA. When the high concentration was combined with the ET-A receptor antagonist BQ123, HA was returned to a level comparable with low concentration ET-1. It can be hypothesized that low concentration ET-1 primarily affects ET-B receptors, which increase HA by increasing nitric oxide (NO) production [25,26,27]. This was corroborated by the abolished increase in HA by the addition of the ET-B receptor antagonist BQ788 to low concentration ET-1. Endothelins are also known to enhance the release of prostaglandins by stimulation of ET-B receptors located on vascular endothelial cells [28,29], which also elevate HA production [27,30,31]. The high concentration of ET-1 may primarily affect ET-A receptors, which have been shown to increase CD44 expression with a BQ123-sensitive mechanism [32]. We have previously demonstrated an inverse relationship between elevated levels of surface CD44 on RMICs and supernatant HA, suggesting increased internalization [11] and thereby reduced levels of HA in the supernatant. When the ET-A receptor antagonist BQ-123 was included in the present study HA was elevated, not only back to control levels, but to the levels corresponding to the low concentration of ET-1, suggesting an action on the still active and intact ET-B receptor. When the ET-B receptor antagonist BQ788 was included, the HA amount no longer differed from the control. The observed changes in HA in the present study fit well with the demonstrated effects of ET-A vs. ET-B receptor activation on medullary fluid handling. ET-B activation reduces fluid reabsorption over NO, while ET-A activation increases fluid reabsorption [33]. This fit well with our previous finding that an intact NO-system is required for the hydration-induced medullary HA-elevation to occur [34].

Ang II and vasopressin in combination reduced the HA content in the supernatant of cultured RMICs and we have previously shown that vasopressin infusion in vivo reduces papillary HA [34], presumably over the V_1_-receptor, since the selective V_2_-receptor agonist desmopressin failed to produce such a response [3], although this may be a concentration issue. The finding of V_1a_ receptors but not of the V_2_ subtype on RMICs [13] corroborates our suggestion. Vasopressin stimulates the activity of Hyal in the rat renal papilla. The activation of these enzymes is associated with a decrease in the content of HA [35]. In homozygous Brattleboro rats lacking vasopressin, the urine osmolality and Hyal activity of renal papillary tissue were closely related after vasopressin treatment [36]. In support of this notion, we have demonstrated that outer medullary HA content is increased in Brattleboro rats, thus inferring reduced breakdown [3]. It has also been demonstrated that antisera against rat kidney Hyal blocks the hydro-osmotic effect of vasopressin [37]. Furthermore, in a study by Ivanova et al. [38], the mRNA expressions of Hyal1 and Hyal2 were increased in the medullary tissue after treatment with a vasopressin analogue. In the present study, vasopressin alone did not reduce HA with statistical significance, only when combined with Ang II. We have, however, in a previous study found a reduction of HA after vasopressin treatment [34]. The discrepancy in results is not clear. The effector mechanism underlying the reduction of HA by Ang II treatment (in combination with vasopressin) seems to be, at least partly, due to increased Hyal activity. This would fit with previous data on neonatal angiotensin converting enzyme (ACE) inhibition showing reduced Hyal1 mRNA expression in the renal medulla and reduced urine Hyal activity early in the newborn rat [39]. It is also noteworthy that these two hormones are simultaneously elevated during dehydration.

In the present study on RMICs, it is clear that the HA content is reduced after high concentration ET-1 (ET-A receptor mediated) and the combination of Ang II and vasopressin. Furthermore, it is well known that both Ang II and vasopressin levels in plasma are elevated during dehydration/antidiuresis (i.e., when medullary HA levels in vivo are reduced). However, previous studies have shown that ET-1, through its action over the ET-A receptor, and Ang II, over the AT_1_ receptor, increase proliferation and extracellular matrix production (ECM) by RMICs in culture [40,41]. What could be the underlying cause for this apparent contradiction? The standard index for ECM production (i.e., ^35^S-methionine/cysteine incorporation) is not a measure of HA production, since HA does not incorporate methionine/cysteine, being that it is a sugar compound. This index is more correct for estimating collagen-related production, and the true relationship between different matrix components has not been demonstrated in parallel with giving the ECM-index. It has, however, been shown that laminin production by RMICs increases after Ang II [41]. A reduced HA content in the medullary interstitium provides favorable conditions for an increase in the permeability of glycosaminoglycan structures adjacent to the cell surface. Since RMICs seem to provide structural support for the renal medulla, it could be speculated that the increase in ECM production, like laminin, would maintain structural integrity when HA levels are reduced in parallel to increase in vivo interstitial water permeability. However, a reduced HA content in parallel with elevation of collagen in a tissue would, during pathological conditions, provide for fibrosis, since the HA reduction leads to reduced viscoelasticity and hydration.

CD44 is the main cell surface receptor for HA [15]. Besides providing a signal response to HA, it participates in HA endocytosis as a scavenger receptor [42]. In the present study, RMIC surface expression of CD44 was reduced by low media osmolality when supernatant HA content was increased, which could provide a pathway for regulation via reduced internalization and degradation. However, in the two other situations when CD44 expression was altered (Ang II + vasopressin and low concentration ET-1, respectively), supernatant HA content changed in an opposite direction, which does not enable a causal relationship. The underlying mechanism to the reduced CD44 expression after Ang II + vasopressin and elevated expression after low concentration of ET-1 is unclear and warrants further investigation.

The Hyal inhibitor used in the present study (l-ascorbic Acid 6-hexadecanoate, Asc-P) is a documented potent inhibitor of different Hyal activities [43]. The importance of Hyal activity for regulating HA turnover in RMICs is clear. When inhibiting Hyal activity during iso-osmotic conditions, supernatant HA increases to similar levels as RMICs grown under hypo-osmotic conditions. As stated above, this would fit well with the suggestion that the elevation in HA during hypo-osmotic conditions occurs through a reduced activity of intracellular Hyals.

Upon continuous hydration in the rat, the medullary HA-levels increase and peak after about 2 h and return to control levels after about 4 h of hydration [2]. Interestingly, peak urine flow rate was also observed after 2 h of hydration. It could be speculated that medullary HA in the rat is primarily important for the acute and rapid exclusion of water during excessive intake.

Dwyer et al. [44] found that in obese rabbits the medullary HA is selectively elevated. It was suggested that a possible distension could occur in the renal medulla with consequences for interstitial hydrostatic pressure and reabsorption. Such a constitutively elevated amount of HA could therefore result in a reduced reabsorptive capacity leading to hypohydration. Whether this can partly explain the higher incidence of hypohydration in obese US adults remains to be established [45].

The concentrations of drugs used in the present study are generally high. However, this was done to achieve maximum response from the systems observed. It is, furthermore, worth noting that the various peptide receptors on RMICs are spatially close and overlapping [46] and complex interactions may therefore occur between components of the intracellular chain of reactions which are transmitted as the hormonal signal. How this affects the results of the present study is unknown.

In conclusion, the present study demonstrates an important regulatory influence of hormones involved in body fluid balance on HA handling by RMICs thereby supporting the concept of a dynamic involvement of interstitial HA on renal fluid handling.

## 4. Materials and Methods

### 4.1. Animals

All animal procedures were approved by the local animal ethics committee at Uppsala University (Approval code C290/11, approval date 25 November, 2011). Male Sprague-Dawley rats weighing 80–90 g (Charles River, Sulzfeld, Germany) were used.

### 4.2. Culture of RMICs

RMICs were isolated from kidneys of young Sprague-Dawley rats as previously described [47,48]. For the first weeks, the culture is primarily epithelial, but after that it consists of a homogenous non-epithelial population. The cells were used for experiments at passage 7 and later. The described isolation and culturing methodology produce cells containing the characteristic lipid vacuoles.

### 4.3. Experimental Protocol—In Vitro

RMICs were plated at a density of about 5 × 10^4^ cells/cm^2^ for 48 h in a 1:1 mixture of two media: Roswell Park Memorial Institute (RPMI) 1640 culture medium and Dulbecco’s Modified Eagle’s Medium (DMEM) culture medium conditioned by 3T3 mouse fibroblasts, the mixture containing a total of 15% fetal bovine serum, as previously described [47]. The cells were then treated for 24 h with different compounds described below. Following 24 h treatment, the supernatant was collected and analyzed for HA content and Hyal activity. Cells were harvested and analyzed for CD44 expression and gene expression of HAS and Hyals. The amount of protein was determined using a routine method (DC Protein Assay, Bio-Rad Laboratories, Hercules, CA, USA). Ang II (Bachem, Bubendorf, Switzerland), vasopressin (Sigma-Aldrich, St. Louis, MO, USA), or a combination of the two were given in concentrations of 10^−6^ M. ET-1 (Sigma-Aldrich) was administrated to provide final concentrations of 10^−10^ M, 10^−8^ M, or 10^−6^ M. The highest ET-1 concentration (10^−6^ M) was also given together with the selective ET-A receptor antagonist BQ123 (10^−6^ M) (Sigma-Aldrich). The lowest ET-1 concentration (10^−10^ M) was also given together with the selective ET-B receptor antagonist BQ788 (10^−6^ M) (Sigma-Aldrich). The Hyal inhibitor l-ascorbic acid 6-hexadecanoate (Asc-P) was purchased from Sigma-Aldrich and administered in a final concentration of 10^−7^ M. The HA synthesis inhibitor 4-methylumbelliferone (4-MU; Sigma-Aldrich) was used in a concentration of 10^−6^ M. Growth media osmolality was reduced to 200 mOsm/kg H_2_O by 2:3 dilution with distilled water.

### 4.4. Analysis of HA

HA content in supernatants from RMICs in culture was measured using a commercially available enzyme-linked immunosorbent assay (Echelon Biosciences Inc., Salt Lake City, UT, USA) by following the enclosed instructions and was then related to the amount of protein.

### 4.5. Hyaluronidase Activity in Supernatants

HA from rooster comb and all reagents for the polymerization of electrophoretic gels were obtained from Wako Pure Chemical Industries (Osaka, Japan), Alcian blue 8GX from Fluka Chemical (Buchs, Switzerland), and Actinase E from Kaken Pharmaceutical (Tokyo, Japan). Supernatant Hyal activity was determined by quantitative zymography [49] with a slight modification because of its very low activity. Briefly, three volumes of supernatant were mixed with one volume of 2× Laemmli sample buffer containing 8% sodium dodecyl sulfate (SDS) and no reducing reagent. A control rat serum used as a standard was diluted 200-fold with 0.15 M NaCl containing 0.1 mg/mL Bovine serum albumin (BSA) and mixed with an equivalent volume of Laemmli sample buffer containing 4% SDS and no reducing reagent. After incubation for 1 h at 37 °C, 32 μL of the supernatant mixture and 2–20 μL of control serum mixture were applied to 7% SDS-polyacrylamide gels containing 0.17 mg/mL HA. After electrophoretic run at 20 mA for approximately 90 min at 4 °C, gels were rinsed with 2.5% Triton X-100 for 80 min at room temperature and incubated with 0.1 M formate buffer (pH 3.5 and containing 0.03 M NaCl) for 24 h at 37 °C on an orbital shaker. Gels were then treated with 0.1 mg/mL Actinase E in 20 mM Tris-HCl buffer (pH 8.0) for 2 h at 37 °C. To visualize digestion of HA, gels were stained with 0.5% Alcian blue in 25% ethanol: 10% acetic acid. After destaining, gels were counterstained with Coomassie brilliant blue R-250. For the determination of Hyal activity, the stained gel was scanned on an Image Scanner (GE healthcare Japan, Tokyo, Japan) and scans were analyzed using Image J 1.42q software (National Institutes of Health, Rockville, MD, USA). The relative band intensity (RI) of supernatant Hyal activity was calculated from the ratio of the band intensity of Hyal activity from 0.05 µL of a control rat serum.

### 4.6. CD44 Analysis

Prior to CD44 analysis by western blot the surface proteins were isolated (Pierce^®^ Cell surface protein isolation kit, Pierce Biotechnology, Rockford, IL, USA). Molecular weight separation was performed on 10% Tris-HCl gels with Tris/glycine/SDS buffer, the proteins transferred to nitrocellulose membranes, and CD44 detected with sheep anti-rat CD44 (0.1 µg/mL; R&D Systems, Minneapolis, MN, USA) and HRP-conjugated rabbit anti-sheep (1:5000; Kirkegaard and Perry Laboratories, Gaithersburg, MD, USA). Luminescent signal was captured on an enhanced chemiluminescence (ECL)-camera system (Kodak image station 2000; New Haven, CT, USA). β-actin was detected with mouse anti-rat β-actin antibody (1:20,000, Sigma-Aldrich, St Louis, MO, USA) and secondary horseradish peroxidase (HRP)-conjugated goat-anti mouse antibody (1:10,000; Kirkegaard and Perry Laboratories, Gaithersburg, MD, USA). CD44 western blot analysis of samples from isolated surface proteins was normalized to the β-actin expression.

### 4.7. Gene Expression Analysis

**Figure 9:**
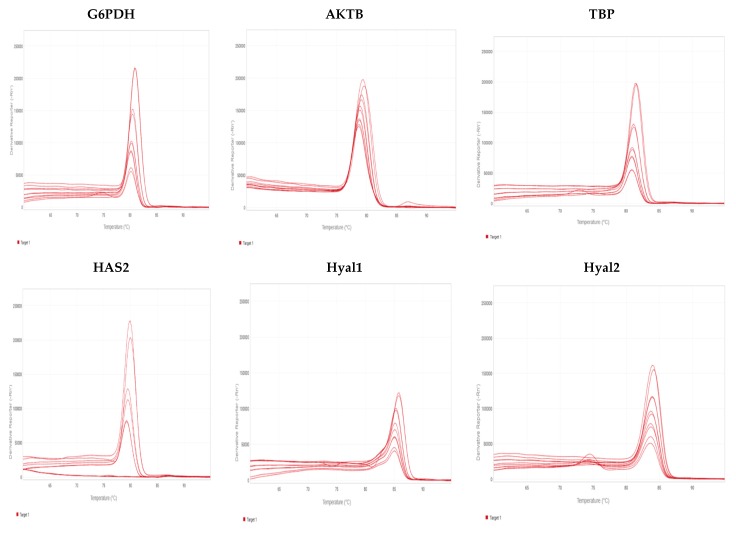
Melt curves for the primers used.

Total RNA was isolated from the cells (RNAquous^®^-4PCR, Ambion, Austin, TX, USA). cDNA was obtained from the RNA (iScript™cDNA Synthesis Kit, Bio Rad Laboratories, Hercules, CA, USA), and the following semi-quantitative real-time PCR was performed by LightCycler^®^ FastStart DNA MasterPLUS SYBR Green I (Roche Diagnostics, Mannheim, Germany) in a Lightcycler system (Roche Diagnostics, Mannheim, Germany). PCR products were verified by agarose gel electrophoresis. The mRNA analyzed were Hyal1 and 2, and HAS2. All values were normalized for reference genes TATA-binding protein (TBP), β-actin (Actb), and glucose-6-phosphate dehydrogenase (G6PDH). Values were then expressed as normalized values for the means of the reference genes by using the formula: 2^Ct(reference genes) – Ct(gene of interest), where Ct is the cycle number and Ct for the reference genes is a mean of the cycle numbers for the reference genes, which did not differ much from each other. Primers were obtained from MWG Biotech (Ebersberg, Germany), with sequences presented in Table 1. Primers were evaluated in terms of efficiency (Table 2), melt curves (Figure 9), and verified product size.

### 4.8. Statistical Analysis

Data are given as mean values ± SEM. The comparison between groups was evaluated with one-way ANOVA followed by Fisher´s Least Significant Difference (LSD) post-hoc test. Parameters presented as percentage in graphs were statistically evaluated using the original values. A *p*-value of <0.05 was considered statistically significant.

## Figures and Tables

**Table 1:** Primer sequences for *HAS2*, *Hyal1*, and *Hyal2*.

Gene	Gene Accession	Forward Primer	Reverse Primer
*G6PDH*	NM_017006.2	GTCATGCAGAACCACCTCCT	ACATACTGGCCAAGGACCAC
*AktB*	NM_031144.3	GCCCTGGCTCCTAGCACC	CCACCAATCCACACAGAGTACTTG
*TBP*	NM_001004198.1	ACCCTTCACCAATGACTCCTATG	ATGATGACTGCAGCAAATCGC
*HAS2*	NM_013153.1	GTGACTGCACCAGTTCCGCTAA	CATGTCTAATGGGACTGCACACAAG
*Hyal1*	NM_207616.1	TCGGACCCTTTATCCTGAAC	TTCTTACACCACTCTCCACTC
*Hyal2*	NM_172040.2	CGTTACGTCAAGGCAGTCAG	AGGTACACGGAGGGAAAGAG

**Table 2:** Primer efficiency for *HAS2*, *Hyal1*, and *Hyal2*.

Gene	Ct Average NOsm (*n* = 8)	Ct Average LOsm (*n* = 8)	Primer Efficiency (%)
*G6PDH*	25.2 ± 0.2	25.5 ± 0.2	96.6
*AKTB*	24.4 ± 0.2	22.5 ± 0.2	98.3
*TBP*	17.1 ± 0.1	17.0 ± 0.2	120.1
*HAS2*	27.3 ± 0.2	29.0 ± 0.3	104.0
*Hyal 1*	27.3 ± 0.4	26.7 ± 0.4	124.5
*Hyal 2*	23.6 ± 0.2	24.3 ± 0.2	125.1

NOsm, normal osmolality; LOsm, low osmolality.

## Abbreviations

HA	Hyaluronan
HAS	Hyaluronan synthase
Hyal	Hyaluronidase
Ang II	Angiotensin II
RMICs	Renomedullary interstitial cells
ET-1	Endothelin-1
